# Supplementary material for: Time-dependent exchange creates the time-frustrated state of matter
Source: Sci Rep. 2022 Sep 28;12:16177. doi: 10.1038/s41598-022-19751-y (PMC9519972; doi:10.1038/s41598-022-19751-y)
Supplement: Supplementary file 1 — Supplementary Information. [file 41598_2022_19751_MOESM1_ESM.pdf]

Time-dependent exchange creates the time-frustrated state of matter Supplementary Information

V. E. Valiulin [1,2], N. M. Chtchelkatchev [1], A. V. Mikheyenkov [1,2] & V. M. Vinokur [3,4,*].

July 21, 2022

[1]Vereshchagin Institute of High Pressure Physics, Russian Academy of Sciences, 108840 Troitsk, Moscow, Russia; [2]Moscow Institute of Physics and Technology, 141701, Dolgoprudny, Russia; [3]Terra Quantum AG, St. Gallerstrasse 16A, CH-9400 Rorschach, Switzerland; [4]Physics Department, City College of the City University of New York, 160 Convent Ave, New York, NY 10031, USA; [*]correspondence to be sent to vv@terraquantum.swiss

Analytical consideration

1. Anisotropic exchange

In the manuscript we focus on the moments coupled via the retarded exchange interaction. For two sites and the isotropic exchange the characteristic equation is set by the determinant (23) of the main text. Here we introduce routinely the spin anisotropy (with the constant ρ) and present the corresponding determinant

$$\Delta(\omega) = \begin{pmatrix} i\omega + \rho\lambda & \rho m_1^0 & -\lambda J(\omega) & -J(\omega) m_1^0 \\ -\rho m_1^0 & i\omega + \rho\lambda & J(\omega) m_1^0 & -\lambda J(\omega) \\ -\lambda J(\omega) & -J(\omega) m_2^0 & i\omega + \rho\lambda & \rho m_2^0 \\ J(\omega) m_2^0 & -\lambda J(\omega) & -\rho m_2^0 & i\omega + \rho\lambda \end{pmatrix} \tag{1}$$

We remind that $(m_1^0)^2 = (m_2^0)^2 = 1$. Note also, the particular form of $J(\omega)$ is not yet set. The exact characteristic equation $\Delta(\omega) = 0$ has the following form:

$$\begin{aligned}\Big[(\rho\lambda + i\omega)^2 + \rho^2\Big]^2 - 2J(\omega)^2\Big[\omega^2 m_1^0 m_2^0 + (\rho\lambda^2 + i\lambda\omega + \rho)^2\Big] \\ + J(\omega)^4\Big(\lambda^2 + 1\Big)^2 = 0\end{aligned} \tag{2}$$

In the limit of small λ equation (2) up to linear terms in λ reads

$$\begin{aligned}\Delta \approx 4i\rho\omega\lambda(\rho^2 - \omega^2 - J^2(\omega)) + J^4(\omega) - 2J^2(\omega)(\rho^2 + \omega^2 m_1^0 m_2^0) \\ + \Big(\rho^2 - \omega^2\Big)^2\end{aligned} \tag{3}$$

which with the adopted form of $J(\omega)$

$$J(\omega) = G\frac{\omega}{\omega + i\omega_0} \tag{4}$$

leads to rather cumbersome expression, it is not presented it here. We consider most-common cases — FM/AFM initial configuration.

FM, $m_1^0 = m_2^0 = +1$

In this case the roots of the characteristic equation are simplified and appear to be the following

$$\omega_{1-4} = \frac{1}{2}\Big(\rho \pm G - i\delta \pm i\sqrt{(-i\rho - iG - \delta)^2 - 4i\rho\delta}\Big)$$

$$\omega_{5-8} = \frac{1}{2}\Big(-\rho \pm G - i\delta \pm i\sqrt{(-i\rho - iG + \delta)^2 + 4i\rho\delta}\Big)$$

though the analysis of stability criteria remains rather complex.

AFM, $m_1^0 = -m_2^0 = +1$

In this case the roots of the characteristic equation are slightly less elegant

$$\begin{aligned}\omega_{1-2} = -\frac{i\delta}{2} - \frac{1}{2}\sqrt{(}\rho^2 - G^2 + \frac{1}{3}D_{-+-} + 2^{1/3}D^2_{+-+}/(3R^{1/3})+ \\ \frac{1}{3\,2^{1/3}}R^{1/3}) - \frac{1}{2}\sqrt{(}D_{+--} + \frac{1}{3}D_{+-+} - 2^{1/3}D^2_{+-+}/(3R^{1/3})- \\ \frac{1}{3\,2^{1/3}}R^{1/3} - (16i\rho^2\delta + 8i\delta^3 + 8i\delta D_{-+-})/(4\sqrt{(}\rho^2 - G^2 + \frac{1}{3}D_{-+-}+ \\ 2^{1/3}D^2_{+-+}/(3R^{1/3}) + \frac{1}{3\,2^{1/3}}R^{1/3})))\end{aligned} \tag{5}$$

$$\begin{aligned}\omega_{3-4} = -\frac{i\delta}{2} - \frac{1}{2}\sqrt{(}\rho^2 - G^2 + \frac{1}{3}D_{-+-} + 2^{1/3}D^2_{+-+}/(3R^{1/3})+ \\ \frac{1}{3\,2^{1/3}}R^{1/3}) + \frac{1}{2}\sqrt{(}D_{+--} + \frac{1}{3}D_{+-+} - 2^{1/3}D^2_{+-+}/(3R^{1/3})- \\ \frac{1}{3\,2^{1/3}}R^{1/3} - (16i\rho^2\delta + 8i\delta^3 + 8i\delta D_{-+-})/(4\sqrt{(}\rho^2 - G^2 + \frac{1}{3}D_{-+-}+ \\ 2^{1/3}D^2_{+-+}/(3R^{1/3}) + \frac{1}{3\,2^{1/3}}R^{1/3})))\end{aligned} \tag{6}$$

$$\begin{aligned}\omega_{5} = -\frac{i\delta}{2} + \frac{1}{2}\sqrt{(}\rho^2 - G^2 + \frac{1}{3}D_{-+-} + 2^{1/3}D^2_{+-+}/(3R^{1/3})+ \\ \frac{1}{3\,2^{1/3}}R^{1/3}) - \frac{1}{2}\sqrt{(}D_{+--} + \frac{1}{3}D_{+-+} - 2^{1/3}D^2_{+-+}/(3R^{1/3})- \\ \frac{1}{3\,2^{1/3}}R^{1/3} + (16i\rho^2\delta + 8i\delta^3 + 8i\delta D_{-+-})/(4\sqrt{(}\rho^2 - G^2 + \frac{1}{3}D_{-+-}+ \\ 2^{1/3}D^2_{+-+}/(3R^{1/3}) + \frac{1}{3\,2^{1/3}}R^{1/3})))\end{aligned} \tag{7}$$

$$\begin{aligned}\omega_{6} = -\frac{i\delta}{2} + \frac{1}{2}\sqrt{(}\rho^2 - G^2 + \frac{1}{3}D_{-+-} + 2^{1/3}D^2_{+-+}/(3R^{1/3})+ \\ \frac{1}{3\,2^{1/3}}R^{1/3}) - \frac{1}{2}\sqrt{(}D_{+--} + \frac{1}{3}D_{-+-} - 2^{1/3}D^2_{-+-}/(3R^{1/3})- \\ \frac{1}{3\,2^{1/3}}R^{1/3} + (16i\rho^2\delta + 8i\delta^3 + 8i\delta D_{-+-})/(4\sqrt{(}\rho^2 - G^2 + \frac{1}{3}D_{-+-}+ \\ 2^{1/3}D^2_{-+-}/(3R^{1/3}) + \frac{1}{3\,2^{1/3}}R^{1/3})))\end{aligned} \tag{8}$$

$$\begin{aligned}\omega_{7-8} = -\frac{i\delta}{2} + \frac{1}{2}\sqrt{(}\rho^2 - G^2 + \frac{1}{3}D_{-+-} + 2^{1/3}D^2_{+-+}/(3R^{1/3})+ \\ \frac{1}{3\,2^{1/3}}R^{1/3}) + \frac{1}{2}\sqrt{(}D_{+--} + \frac{1}{3}D_{+-+} - 2^{1/3}D^2_{+-+}/(3R^{1/3})- \\ \frac{1}{3\,2^{1/3}}R^{1/3} + (16i\rho^2\delta + 8i\delta^3 + 8i\delta D_{-+-})/(4\sqrt{(}\rho^2 - G^2 + \frac{1}{3}D_{-+-}+ \\ 2^{1/3}D^2_{+-+}/(3R^{1/3}) + \frac{1}{3\,2^{1/3}}R^{1/3})))\end{aligned} \tag{9}$$

where

$$R = K + \sqrt{-4D_{-+-}^{6} + K^2}; \tag{10}$$

$$K = -108\rho^4\delta^2 - 108\rho^2\delta^4 - 108\rho^2\delta^2 D_{-+-} + 2D_{-+-}^{3} \tag{11}$$

and

$$D_{\pm\pm\pm} = \pm\rho^2 \pm G^2 \pm \delta^2 \tag{12}$$

2. Isotropic non-frustrated exchange Here we consider the non-frustrated isotropic exchange (with non-zero time average)

$$\int_{-\infty}^{t\to\infty} J(t-\tau)d\tau = J_0 \neq 0 \tag{13}$$

The corresponding determinant is the following

$$\Delta(\omega) = \begin{pmatrix} i\omega + J_0\lambda r & J_0 m_2^0 & -\lambda J(\omega) & -J(\omega)m_1^0 \\ -J_0 m_2^0 & i\omega + J_0\lambda r & J(\omega)m_1^0 & -\lambda J(\omega) \\ -\lambda J(\omega) & -J(\omega)m_2^0 & i\omega + J_0\lambda r & J_0 m_1^0 \\ J(\omega)m_2^0 & -\lambda J(\omega) & -J_0 m_1^0 & i\omega + J_0\lambda r \end{pmatrix}$$

where $r = m_1^0 m_2^0$ ($r = \pm 1$ for FM/AFM cases). Note also, the particular form of $J(\omega)$ is not yet set.

$$\begin{aligned} \Delta(\omega) &= [J^2(\omega)\left(\lambda - im_1^0\right)\left(\lambda - im_2^0\right) \\ &+ \left(\omega - J_0\left(1 + i\lambda m_1^0\right)m_2^0\right)\left(\omega - J_0\left(1 + i\lambda m_2^0\right)m_1^0\right)] \times \\ & [J^2(\omega)\left(\lambda + im_1^0\right)\left(\lambda + im_2^0\right) \\ &+ \left(\omega + J_0\left(1 - i\lambda m_1^0\right)m_2^0\right)\left(\omega + J_0\left(1 - i\lambda m_2^0\right)m_1^0\right)] \end{aligned}$$

Formally the equation $\Delta(\omega) = 0$ has two doubly degenerate roots until the $J(\omega)$ dependence is not fleshed out

$$\begin{aligned} \omega_{1-2} &= \frac{1}{2}(\pm J_0(m_1^0 + m_2^0) + 2i\lambda J_0 m_1^0 m_2^0 \\ &- \sqrt{-4J^2(\omega)\left(\lambda + im_1^0\right)\left(\lambda + im_2^0\right) + 2J_0^2\left(1 - m_1^0 m_2^0\right)}) \end{aligned}$$

For FM case $m_1^0 = m_2^0 = +1$ this leads to

$$\omega_{1-2} = J_0(\pm 1 + i\lambda) - \sqrt{-(i+\lambda)^2 J^2(\omega)}$$

For AFM $m_1^0 = -m_2^0 = +1$

$$\omega = -iJ_0\lambda \pm \sqrt{J_0^2 - (1+\lambda^2)\,J^2(\omega)}$$

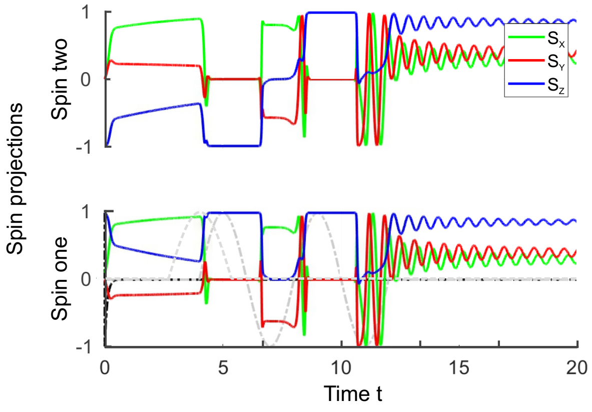

Figure 1 | (Color online) Perturbation in the form of alternating pulses sequence successively converts FM to AFM and vise versa. The Landau-Lifshitz-Gilbert equation parameters for both panels are $\gamma = 1$, $\lambda = 1$, the retarded exchange parameters are $G = 10$, $\omega_0 = 10$. Black dash-dotted lines — exchange potential, the retardation is almost invisible in the adopted time scale. Perturbation — two periods of sinusoid $A\sin(\alpha t)$ with $A = 50$. Spin projections and exchange scales differ.

Analogously to the previous section, the stability criterion for FM/AFM stationary configurations could be obtained with a given form of $J(t)$ ($J(\omega)$), and J_0. It is easy to show, that in the limit of constant exchange the results boil down to common textbook expressions.

Numerical consideration

1. Consequent spin order switch In the main text it is shown that in the absence of anisotropy half-sinusoidal pulse of the external magnetic field switches the FM state into the AFM one, however for $t \to \infty$ the system returns to initial FM. Here we demonstrate the effect is even more peculiar — the external magnetic field pulse alters the system spin order form FM to AFM and vise versa, the $t \to \infty$ state returning to initial FM (See Fig. 1).

2. The influence of exchange parameters on the final state Fig. 3 of the main text demonstrates time evolution of the initial state for different values of ω_0. We remind that two magnetic moments are considered, the exchange interaction is $J(\omega) = G\,\omega/(\omega + i\omega_0)$. The effect is the following. For $\omega_0 \lesssim 7.5$ (relatively slow retardation) the final stable state is FM, for $\omega_0 \gtrsim 10$ (relatively fast retardation) the final stable state is AFM.

For more complex system of four magnetic moments the effect is qualitatively the same (See Fig. 2). Relatively slow retardation leads to FM final stable state, relatively fast retardation results in AFM final stable state.

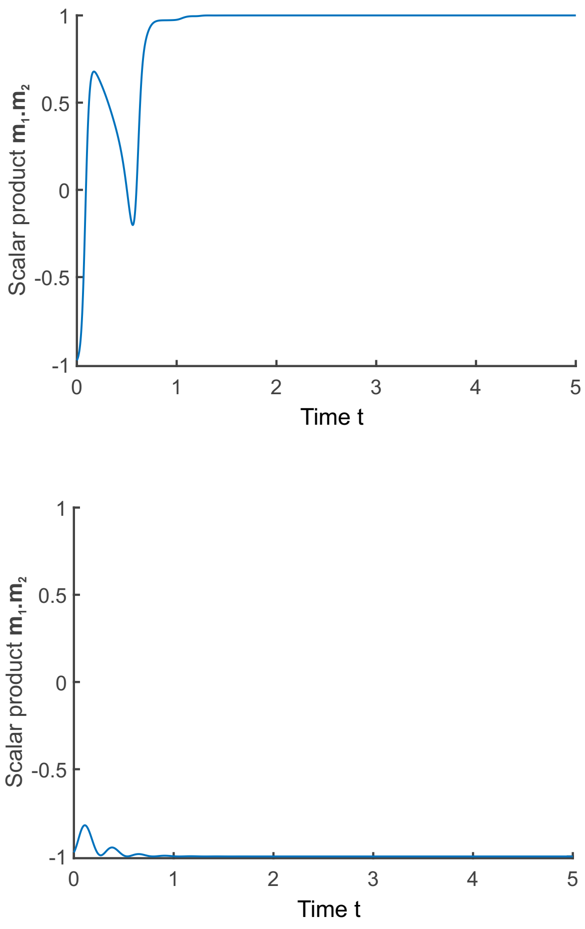

Figure 2 | The figure demonstrates time evolution of the mean scalar product (four magnetic moments) for different values of ω_0. For small $\omega_0 = 10$ (slow retardation, upper panel) the final stable state is FM, for large $\omega_0 = 30$ (fast retardation, lower panel) the final stable state is AFM. The Landau-Lifshitz-Gilbert equation parameters are $\gamma = 1$, $\lambda = 1$, anisotropy parameter $\rho = 5$.

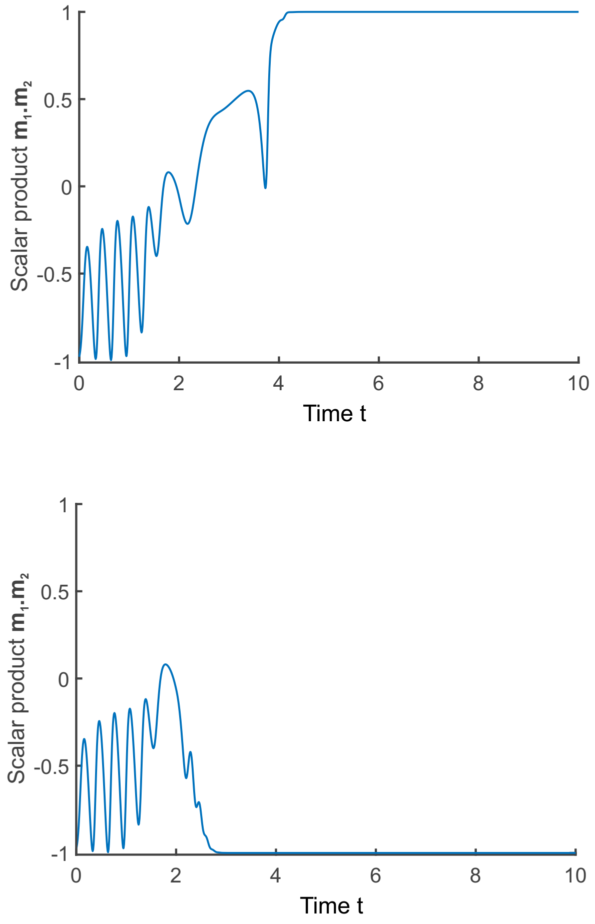

Figure 3 | The figure demonstrates possibility to manipulate the final stable sate by the form of external magnetic pulse. Mean scalar product (four magnetic moments) is shown. For $A = -15$ (negative pulse, upper panel) the final stable state is FM, for $A = 30$ (positive pulse, lower panel) the final stable state is AFM. The Landau-Lifshitz-Gilbert equation parameters are $\gamma = 1$, $\lambda = 1$, anisotropy parameter $\rho = 5$, the retarded exchange amplitude is $G = 10$, $\omega_0 = 30$.

3. The influence of the perturbation on the final state Fig. 4 of the main text demonstrates that the form of magnetic pulse allows to manipulate the final stable sate. For half sinusoid $A\sin(\alpha t)$ with $A \lesssim -15$ (negative pulse) the final stable state is FM, for $A \gtrsim 5$ (positive pulse) the final stable state is AFM.

For more complex system of four magnetic moments the effect is qualitatively the same (See Fig. 3). Negative pulse leads to FM final stable state, positive pulse results in AFM final stable state.

Application

1. Four-bit memory cell Modern computing devices are based on simple binary logic and suitable hardware. However, the cutting edge of information technologies and the immense data flows require new soft- and hardware types. The promising decision here is to implement non-binary (Many-Valued) logic and appropriate machinery (Refs. [3–6]). One possible way is to exploit the devices with both magnetic and electric degrees of freedom, e.g., Ref. [1]), (See Fig. 4).

As it is discussed in the main text, all four states (a-d) in the computer memory element *magnet-ferroelectric-magnet* with time frustrated exchange have the same magnetic energy — due to the effect of dynamic frustration studied here that ensures stability of both AFM and FM configurations. Thin layer of ferroelectric not only transmits current but also provides an exchange interaction. Left and right magnetic metals are made from different materials and so left (right), up(down) densities of states, $\nu_{L(R)}^{up(dn)}$, at Fermi level are essentially different. As the result, electrical resistance is different in (a)-(d) [1,2]. It is defined by the expression:

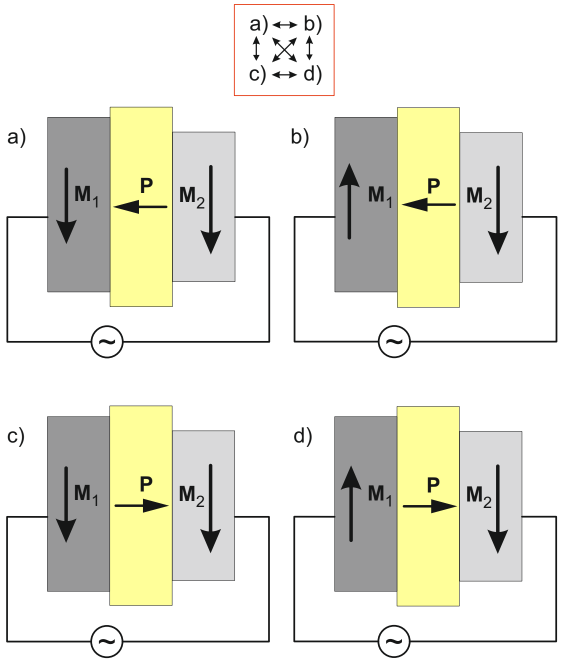

Figure 4 | **All four states (a-d) in the computer memory element concept (see, e.g., Ref. [1]) have the same magnetic energy due to the effect of dynamic frustration studied here that ensures stability of both AFM and FM configurations.** Thin layer of ferroelectric not only transmits current but also provides an exchange interaction between the ferromagnets. Left and right ferromagnetic metals are made from different materials and so left (right), up(down) densities of states, $\nu_{L(R)}^{up(dn)}$, at Fermi level are essentially different. As the result, electrical resistance is different in (a)-(d) [1,2]. One can switch between all the states (a)-(d) by applied voltage (reverse direction of **P**) and (or) local magnetic field.

$$R(M_1, M_2, P) \propto |t_{\mathbf{P}}|^2\, \mathrm{DOS}_{E_F}^{(M_1,\mathrm{up(dn)})} \mathrm{DOS}_{E_F}^{(M_2,\mathrm{up(dn)})} \tag{14}$$

where $\mathrm{DOS}_{E_F}^{(M_i,\mathrm{up(dn)})}$, $i = 1, 2$ is the up(down) density of states at the Fermi-level of left and right ferromagnetes and $t_{\mathbf{P}}$ is the tunnel amplitude that depends on the direction of ferroelectric polarization. Thus all the four states (a)-(d) are distinguishable by the resistivity $R(M_1, M_2, P)$.

It is also important that one can switch between all the states (a)-(d) by applied voltage (reverse direction of **P**) and (or) local magnetic field.

References

1. Garcia, V. & Bibes, M. Ferroelectric tunnel junctions for information storage and processing. *Nat. Commun.* **5**, 4289 (2014).
2. Zhuravlev, M. Y., Sabirianov, R. F., Jaswal, S. S. & Tsymbal, E. Y. Giant electroresistance in ferroelectric tunnel junctions. *Phys. Rev. Lett.* **94**, 246802 (2005).
3. Omori, H. & Wansing, H. An Invitation to New Essays on Belnap-Dunn Logic. In Omori, H. & Wansing, H. (eds.) *New Essays on Belnap-Dunn Logic*, Synthese Library, 1–9 (Springer International Publishing, Cham, 2019).
4. Ceze, L., Nivala, J. & Strauss, K. Molecular digital data storage using dna. *Nat. Rev. Genet.* **20** (2019).
5. Lukyanchuk, I. *et al.* High-symmetry polarization domains in low-symmetry ferroelectrics. *Nano letters* **14** (2013).
6. Baudry, L., Lukyanchuk, I. & Vinokur, V. M. Ferroelectric symmetry-protected multibit memory cell. *Sci. Rep.* **7**, 42196 (2017).
